# Immunochip analysis identifies association of the *RAD50/IL13* region with human longevity

**DOI:** 10.1111/acel.12471

**Published:** 2016-03-22

**Authors:** Friederike Flachsbart, David Ellinghaus, Liljana Gentschew, Femke‐Anouska Heinsen, Amke Caliebe, Lene Christiansen, Marianne Nygaard, Kaare Christensen, Hélène Blanché, Jean‐François Deleuze, Céline Derbois, Pilar Galan, Carsten Büning, Stephan Brand, Anette Peters, Konstantin Strauch, Martina Müller‐Nurasyid, Per Hoffmann, Markus M. Nöthen, Wolfgang Lieb, Andre Franke, Stefan Schreiber, Almut Nebel

**Affiliations:** ^1^ Institute of Clinical Molecular BiologyKiel UniversityKielGermany; ^2^ Institute of Medical Informatics and StatisticsKiel UniversityKielGermany; ^3^ Epidemiology, Biostatistics and BiodemographyDepartment of Public HealthUniversity of SouthernDenmarkOdenseDenmark; ^4^ Department of Clinical GeneticsOdense University HospitalOdenseDenmark; ^5^ Department of Clinical Biochemistry and PharmacologyOdense University HospitalOdenseDenmark; ^6^ Fondation Jean Dausset‐Centre du Polymorphisme Humain (CEPH)ParisFrance; ^7^ Centre National de Génotypage CNG‐IG‐CEAEvryFrance; ^8^ Université Sorbonne Paris Cité‐URENUnité de Recherche en Epidémiologie Nutritionnelle; U557 Inserm; U1125 Inra; Cnam; Université Paris 13CRNH IdFBobignyFrance; ^9^ Department of Gastroenterology, Hepatology and EndocrinologyCharitéCampus MitteBerlinGermany; ^10^ Department of Medicine II – GrosshadernLudwig‐Maximilians‐University MunichMunichGermany; ^11^ Institute of Epidemiology IIHelmholtz Zentrum München – German Research Center for Environmental HealthNeuherbergGermany; ^12^ DZHK (German Centre for Cardiovascular Research), Partner Site Munich Heart AllianceMunichGermany; ^13^ German Center for Diabetes ResearchNeuherbergGermany; ^14^ Institute of Genetic EpidemiologyHelmholtz Zentrum München – German Research Center for Environmental HealthNeuherbergGermany; ^15^ Institute of Medical Informatics, Biometry and EpidemiologyChair of Genetic EpidemiologyLudwig‐Maximilians‐University MunichMunichGermany; ^16^ Department of Medicine ILudwig‐Maximilians‐University MunichMunichGermany; ^17^ Institute of Human GeneticsUniversity of BonnBonnGermany; ^18^ Department of Genomics, Life and Brain CenterUniversity of BonnBonnGermany; ^19^ Division of Medical GeneticsUniversity Hospital Basel and Department of BiomedicineUniversity of BaselBaselSwitzerland; ^20^ Institute of Epidemiology and Popgen BiobankKiel UniversityKielGermany; ^21^ Clinic for Internal Medicine IUniversity Hospital of Schleswig‐HolsteinKielGermany

**Keywords:** 5q31.1, genetic association, human longevity, *IL13*, Immunochip, *RAD50*

## Abstract

Human longevity is characterized by a remarkable lack of confirmed genetic associations. Here, we report on the identification of a novel locus for longevity in the *RAD50/IL13* region on chromosome 5q31.1 using a combined European sample of 3208 long‐lived individuals (LLI) and 8919 younger controls. First, we performed a large‐scale association study on 1458 German LLI (mean age 99.0 years) and 6368 controls (mean age 57.2 years) by targeting known immune‐associated loci covered by the Immunochip. The analysis of 142 136 autosomal single nucleotide polymorphisms (SNPs) revealed an Immunochip‐wide significant signal (*P*_I__mmunochip_ = 7.01 × 10^–9^) for the SNP rs2075650 in the *TOMM40*/*APOE* region, which has been previously described in the context of human longevity. To identify novel susceptibility loci, we selected 15 markers with *P*_I__mmunochip_ < 5 × 10^–4^ for replication in two samples from France (1257 LLI, mean age 102.4 years; 1811 controls, mean age 49.1 years) and Denmark (493 LLI, mean age 96.2 years; 740 controls, mean age 63.1 years). The association at SNP rs2706372 replicated in the French study collection and showed a similar trend in the Danish participants and was also significant in a meta‐analysis of the combined French and Danish data after adjusting for multiple testing. In a meta‐analysis of all three samples, rs2706372 reached a *P*‐value of *P*_I__mmunochip+Repl_ = 5.42 × 10^−7^ (OR = 1.20; 95% CI = 1.12–1.28). SNP rs2706372 is located in the extended *RAD50/IL13* region. *RAD50* seems a plausible longevity candidate due to its involvement in DNA repair and inflammation. Further studies are needed to identify the functional variant(s) that predispose(s) to a long and healthy life.

Despite more than 20 years of research into the genetic basis of human longevity, only alleles in the *APOE* and *FOXO3* genes have repeatedly been shown to be associated with survival to very advanced ages (Schächter *et al*., 1994; Willcox *et al*., 2008; Flachsbart *et al*., 2009; Soerensen *et al*., 2010; Deelen *et al*., 2013). *APOE* and *FOXO3* were initially detected in candidate‐driven case–control investigations, but *APOE* has since then been confirmed in a number of genome‐wide association studies (GWAS). In addition, a single nucleotide polymorphism (SNP) on chromosome 5q33.3 was recently identified in a GWAS meta‐analysis on long‐lived individuals (LLI) aged ≥ 90 years (Deelen *et al*., 2014). Besides the detection of *APOE* and the 5q33.3 locus, longevity GWAS have been relatively unsuccessful and have failed to reveal novel associations with genome‐wide significance or sufficient reproducibility (Deelen *et al*., 2011, 2014; Nebel *et al*., 2011). Here, we performed a large‐scale candidate gene study by targeting established immune‐associated loci present on the Immunochip (Trynka *et al*., 2011). The Immunochip was designed to perform fine‐mapping of GWAS loci of major immune‐mediated diseases using data from the 1000 Genomes Project and other sequencing initiatives. The application of the array in this study is based on the hypothesis that a well‐functioning immune system and efficient anti‐inflammatory networks are potent longevity‐assurance mechanisms (Franceschi *et al*., 2007). We employed the Immunochip to screen 1458 German LLI and 6368 younger controls in a discovery phase (panel A in Table S1) for novel longevity loci followed by replication in 1750 LLI and 2551 younger controls from France and Denmark (panel B in Table S1).

After applying conservative and established quality filters to the German longevity sample, 142 136 autosomal SNPs were available for association analysis (see Appendix S1). We used a predefined threshold of *P *=* *6.15 × 10^−7^ to define statistical significance for the Immunochip‐wide analysis, based on the Bonferroni correction for the number of linkage disequilibrium (LD)‐independent markers on the Immunochip (see Appendix S1). The comparison of the case–control frequencies yielded an Immunochip‐wide significant association signal for the SNP rs2075650 in the *TOMM40*/*APOE* region (*P*_Immunochip_ = 7.01 × 10^−9^; OR = 0.69; 95% CI = 0.60–0.78; Table 1, Figs S1 and S2a). This SNP is in moderate LD (*r* ^2^ = 0.52) with SNP rs429358 that defines the well‐established *APOE* ε4 allele (Deelen *et al*., 2011), and hence, rs2075650 was not considered for replication. With regard to the *FOXO3* and the 5q33.3 loci, the Immunochip is uninformative due to poor coverage (Fig. S2b,c). Eighty‐four other SNPs showed *P*_Immunochip_ < 5 × 10^−4^ in the discovery Immunochip analysis (Table S2). Of these, we selected 15 markers for replication, each one representing the best SNP for a specific associated region defined by the clumping procedure (see Appendix S1, Table S3). The replication analysis was performed in two longevity samples from France and Denmark (France: 1257 LLI and 1811 controls; Denmark: 493 LLI and 740 controls; panel B in Table S1). The signal at SNP rs2706372, located in a region encompassing *RAD50* (radiation sensitive, *Saccharomyces cerevisiae* homolog) and *IL13* (Interleukin 13), replicated in the French sample (*P*_Repl‐France_ = 2.69 × 10^−3^; OR = 1.21; 95% CI = 1.07–1.38; *P*_Repl‐France; adj_ = 0.04 (corrected for 15 tests)) (Table S4). In the smaller Danish sample, the allelic effect of rs2706372 showed a similar trend (*P*_Repl‐Denmark_ = 0.08; OR = 1.19; 95% CI = 0.98–1.45; *P*_Repl‐Denmark; adj_ = 1 (corrected for 15 tests)). In the combined French‐Danish replication sample, meta‐analysis association analysis yielded a *P*‐value of 4.95 × 10^−4^ (OR = 1.21; 95% CI = 1.09–1.34; *P*_Repl‐France‐Denmark; adj_ = 0.0074 (corrected for 15 tests)). In a meta‐analysis of the German discovery and French‐Danish replication samples, rs2706372 reached a *P*‐value of *P*_Immunochip+Repl_ = 5.42 × 10^−7^ (Table 1). Estimates of odds ratios for rs2706372 were consistent across all three studies (OR_Germany_ = 1.19; OR_Denmark_ = 1.19; OR_France_ = 1.21; statistical metric of heterogeneity *I* ^*2*^ = 0.0), supporting the validity of the association finding.

**Table 1:** Immunochip loci associated with human longevity. 5q31.1 (*RAD50*/*IL13*) is a newly associated locus

Chr.	Association boundaries (kb)	dbSNP ID	A1	A2	AF cases	AF controls	Functional annotation	Key genes (*n* additional genes within locus)	Discovery Immunochip (1458/6368)		Replication (1750/2551)		Immunochip + Replication (3208/8919)		
									*P*	OR (95% CI)	*P*	OR (95% CI)	*P*_combined_	OR (95% CI)	*I* ^2^
19q13.32	45357–45444	rs2075650	G	A	0.1080	0.1488	*TOMM40* (intronic)	*TOMM40/APOE* (*3*)	7.01 × 10^−9^	0.69 (0.60–0.78)	a	–	–	–	–
5q31.1	131784–132143	rs2706372	T	C	0.2565	0.2241	*RAD50* (intronic)	*RAD50/IL13* (*7*)	3.11 × 10^−4^	1.19 (1.08–1.31)	4.95 × 10^−4^	1.21 (1.09–1.34)	5.42 × 10^−7^	1.20 (1.12–1.28)	0.0

a SNP rs2075650 was not considered for replication in this study, because the association at 19q31.32 was already established in the French sample (Schächter *et al*., 1994) and parts of the Danish sample (Soerensen *et al*., 2010).

**Chr**: chromosome of marker; **association boundaries**: association boundaries for each index SNP (see Appendix S1). Genomic positions were retrieved from NCBI's dbSNP build v141 (genome build hg19); **dbSNP id:** rs ID of the index SNP; **A1:** minor allele; **A2:** major allele; **AF:** allele frequency of A1 estimated from Immunochip (German population); **key genes:** candidate gene(s) in the region; ***P*** **/OR:** *P*‐value and corresponding allelic odds ratio and 95% confidence interval with respect to A1. We used a significance level of 6.15 × 10^−7^ for the statistical association analysis of the Immunochip in the discovery experiment and in the combined experiment of discovery and replication based on Bonferroni correction (see Appendix S1). For each panel, numbers of LLI/controls are displayed in parentheses; ***I*** ^**2**^ **:** statistical metric of heterogeneity. *I* ^*2*^ ranges from 0 to 100% and is considered low for values 0–25%.

**Associations with other traits:** Overlaps with other disease phenotypes (listed if anywhere within association boundaries, see Appendix S1).

rs2075650: age‐related macular degeneration; Alzheimer's disease; apolipoprotein levels; blood metabolite; brain imaging; C‐reactive protein; cardiovascular disease; carotid intima media thickness; cerebrospinal AB1‐42 levels; cholesterol total; cognitive decline; HDL cholesterol; LDL cholesterol; lipid traits; lipid metabolism; lipoprotein‐associated phospholipase A2 activity and mass; metabolic syndrome; metabolic levels; quantitative traits; response to statin therapy (LDL‐C); sphingolipid levels; triglycerides.

rs2706372: asthma; asthma (childhood, severe); asthma (sex interaction); atopic dermatitis; C‐reactive protein; Crohn's disease; eosinophil counts; fibrinogen; Hodgkin's lymphoma; IgE levels; platelet counts; psoriasis; self‐reported allergy.

Our targeted immune gene approach on a combined European sample of 3208 LLI and 8919 controls resulted in the identification of a novel association for longevity in the *RAD50/IL13* region on chromosome 5q31.1. The lead SNP rs2706372 is located in the intronic region of the *RAD50* gene and is in strong LD with other associated SNPs close to *IL13* and *IL5*. The actual association signal extends even further to include additional genes (Fig. 1). At this point, this observation renders it difficult to assess which gene is actually affected by the association, although *RAD50* is a plausible candidate. The protein encoded by *RAD50* is highly similar to *Saccharomyces cerevisiae* Rad50 which is involved in repairing DNA double‐strand breaks. Similarly, the human RAD50 is integrated in a functional DNA‐binding complex (Kinoshita *et al*., 2015) that is important for recombination, repair, and genomic stability (Trujillo *et al*., 1998). Hence, it is conceivable that variation in *RAD50* could positively influence longevity by increasing DNA stability. Alternatively, it could exert its effect via the direct modulation of cytokine expression; recent evidence suggests at least two possible avenues. First, in dendritic cells RAD15 was found to activate—upon sensing viral DNA—the transcription factor NF‐κB, thus leading to the production of pro‐inflammatory IL‐1β (Roth *et al*., 2014). Second, the *RAD50* gene harbors at its 3’ end an evolutionarily highly conserved locus control region (LCR; Lee *et al*., 2003; Li *et al*., 2010) that regulates the expression of the neighboring cytokine genes *IL‐4*,* IL‐13,* and *IL‐5* (Fig. 1) in Th2 cells (Kelly & Locksley, 2000). Variants in the LCR were found to be associated with asthma (Li *et al*., 2010). Taken together, these findings indicate that the *RAD50* locus may very well contribute to longevity via its role in inflammation and immunity. Nevertheless, it is still possible that the *RAD50* signal is a result of its LD with other markers within the observed association boundaries. Multiple SNPs in the extended *RAD50/IL13* region were previously identified as susceptibility factors for various chronic inflammatory diseases such as Crohn's disease, psoriasis, asthma, and atopic dermatitis (Rioux *et al*., 2000; Li *et al*., 2008, 2010; Paternoster *et al*., 2011). Further studies are therefore needed to identify the functional variant(s) and the underlying molecular mechanisms that predispose(s) to a long and healthy life.

**Figure 1:**
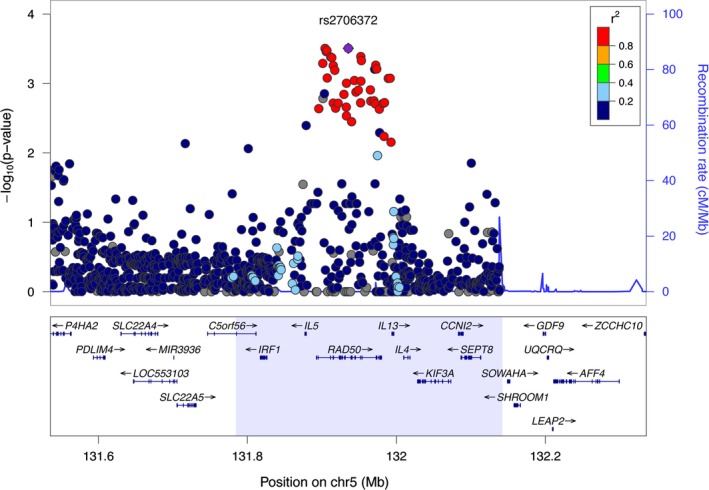
Association plot for 5q31.1 (*RAD50*/*IL13*). Blue shaded region corresponds to locus association boundaries (Table 1 and Appendix S1). Shown are the ‐log_10_ *P‐*values from the Immunochip analysis (*P*_Immunochip_) of the German longevity discovery panel (panel A in Table S1) with regard to the physical location of markers. **Purple diamond:** lead SNP;** filled circles:** analyzed SNPs where the fill color corresponds to the strength of linkage disequilibrium (*r* ^2^) with the lead SNP (for color coding see legend in the upper right corner of the plot); **blue line:** recombination intensity (cM/Mb). Positions and gene annotations are according to NCBI's build 37 (hg19). Plot was generated using LocusZoom (Pruim *et al*., 2010).

## Funding

This study was supported by the RESOLVE project (grant FP7‐HEALTH‐F4‐2008‐202047 to S.S., A.N., and F.F); the Deutsche Forschungsgemeinschaft (DFG) Cluster of Excellence ‘Inflammation at Interfaces’ (grant to A.N. with fellowship for L.G.); the German Federal Ministry of Education and Research (BMBF) within the framework of the e:Med research and funding concept (SysInflame grant 01ZX1306A); the biobank popgen (the popgen 2.0 network is financed by the German Ministry for Education and Research (grant 01EY1103 to W.L.)); the German Federal Ministry for Education and Research within the context of the National Genome Research Network 2 (NGFN‐2), the National Genome Research Network plus (NGFNplus) and the Integrated Genome Research Network (IG) MooDS (grant 01GS08144 to M.M.N., grant 01GS08147); the INTERREG4A program Syddanmark‐Schleswig‐K.E.R.N (with EU funds from the European Regional Development Fund, grant to K.C., A.N., L.C., and F.F.); the VELUX Foundation; the Danish National Research Foundation; the US National Institutes of Health—National Institute on Aging (grant number P01 AG08761 to K.C. and L.C.); the Danish Agency for Science, Technology and Innovation (grant number 09–070081 to K.C. and L.C.). The CEPH centenarian cohort was supported by the “Ministère de l'Enseignement supérieur et de la Recherche”. The SU.VI.MAX cohort was funded by the French Institut National de la Santé et de la Recherche Médicale, the Institut National de la Recherche Agronomique, the Université Paris 13. The genotyping of French samples was supported by the Commissariat à l'Energie Atomique‐Centre National de Génotypage. The KORA research platform (KORA, Cooperative Research in the Region of Augsburg) was initiated and financed by the Helmholtz Zentrum München—German Research Center for Environmental Health, which is funded by the German Federal Ministry of Education and Research and by the State of Bavaria. Furthermore, KORA research was supported within the Munich Center of Health Sciences (MC Health), Ludwig‐Maximilians‐University Munich, as part of LMUinnovativ. S. B. is supported by DFG grant BR1912/6‐1 and the Else‐Kröner‐Fresenius‐Stiftung (stipend 2010_EKES.32). M. M. N. received support from the Alfried Krupp von Bohlen und Halbach‐Stiftung. The HNR study is supported by the Heinz Nixdorf Foundation (Germany). Additionally, the study is funded by the German Ministry of Education and Science and the German Research Council (DFG; Project SI 236/8‐1, SI236/9‐1, ER 155/6‐1). A. F. receives an endowment professorship by the Foundation for Experimental Medicine (Zurich, Switzerland).

## Conflict of interest

The authors declare no competing financial interests.

## Author contributions

F.F., A.N., A.F., D.E., and S.S. designed research; W.L., A.F., S.S., C.B., S.B., A.P., K.S., M.M.‐N., P.H., and M.M.N. were involved in recruitment of German study subjects and assembling of phenotypic data; F.F. and A.N. organized chip genotyping of German long‐lived individuals; H.B., J.D., C.D., P.G., L.C., M.N., and K.C. performed replication experiments; F.‐A.H., M.N., and C.D. helped with the experimental work; D.E., L.G., A.C., and F.F. analyzed data; D.E., F.F., and A.N. interpreted the data and wrote the manuscript; all authors performed critical revision and approved the final version of the manuscript.

## Supporting information


**Appendix S1** Experimental procedure.
**Table S1** LLI/control panels used in the analysis.
**Table S2** Immunochip association statistics in German panel (panel A in Supplementary Table 1) for 84 SNPs with *P*_Immunochip_ < 5 × 10^−4^.
**Table S3** Immunochip association statistics in German panel (panel A in Supplementary Table 1) for the 15 SNPs selected for replication.
**Table S4** Association statistics in French and Danish samples (panel B in Supplementary Table 1) for the 15 SNPs selected for replication.
**Fig. S1** Manhattan plot of Immunochip association statistics of 142 136 SNPs.
**Fig. S2** Regional association plots (from Immunochip analysis; panel A in Supplementary Table 1) of established longevity susceptibility loci.
**Fig. S3** Principal component analysis of QCed Immunochip data.
**Fig. S4** Quantile‐quantile (Q‐Q) plot for the discovery panel (panel A in Supplementary Table 1).
