# Supplementary material for: Immunochip analysis identifies association of the RAD50/IL13 region with human longevity
Source: Aging Cell. 2016 Mar 22;15(3):585–8. doi: 10.1111/acel.12471 (PMC4854908; doi:10.1111/acel.12471)
Supplement: Supplementary file 1 — Appendix S1 Experimental procedure. Table S1 LLI/control panels used in the analysis. Table S2 Immunochip association statistics in German panel (panel A in Supplementary Table 1) for 84 SNPs with P Immunochip < 5 × 10^−4^. Table S3 Immunochip association statistics in German panel (panel A in Supplementary Table 1) for the 15 SNPs selected for replication. Table S4 Association statistics in French and Danish samples (panel B in Supplementary Table 1) for the 15 SNPs selected for replication. Fig. S1 Manhattan plot of Immunochip association statistics of 142 136 SNPs. Fig. S2 Regional association plots (from Immunochip analysis; panel A in Supplementary Table 1) of established longevity susceptibility loci. Fig. S3 Principal component analysis of QCed Immunochip data. Fig. S4 Quantile‐quantile (Q‐Q) plot for the discovery panel (panel A in Supplementary Table 1). [file ACEL-15-585-s001.docx]

**Supporting Information**

**Experimental procedure**

**Study subjects**

*German Immunochip data set.* A case sample of 1,535 German LLI (male/female ratio approximately 1/3; age-range 94–110 years; mean age 99.0 years) as described previously ([Nebel *et al.* 2005](#_ENREF_8); [Flachsbart *et al.* 2009](#_ENREF_3); [Nebel *et al.* 2011](#_ENREF_9)) was investigated for this study (panel A in **Supplementary Table 1**). The control sample comprised 6,502 younger individuals drawn from German population-based collections including 2,567 Germans from the popgen biorepository ([Krawczak *et al.* 2006](#_ENREF_5)), 1,916 adults from the KORA study (Collaborative Health Research in the Region of Augsburg) ([Holle *et al.* 2005](#_ENREF_4)), 313 individuals recruited by the Bavarian Red Cross, and 206 participants recruited by the Charité-Universitätsmedizin Berlin. Additional samples (1,500) were obtained from the population-based epidemiological Heinz Nixdorf Recall study ([Schmermund *et al.* 2002](#_ENREF_12)). DNA samples were genotyped using the Immunochip custom chip ([Trynka *et al.* 2011](#_ENREF_13)), an Illumina targeted high-density genotyping array with comprehensive coverage of 196,524 SNPs located in 186 autoimmune disease-associated loci. All subjects gave written informed consent to participate in the study. Approval for the project was obtained from the respective review boards.

*French replication sample.* French centenarians were recruited when they were in their 100th year or beyond (Blanché *et al.* 2001). French siblings were recruited when at least two siblings fulfilling age criteria of 90 years or older were alive in a family. The oldest sibling was selected for the association study. The mean age of centenarians and unrelated siblings was 104 and 100 years, respectively (age at death or age at last contact). In total 1,264 elderly were included in the study (male/female ratio approximately 1/4.5, mean age 102.4 years; age-range 91-115+ years). All subjects signed a written informed consent form in accordance with the local review board. The 1,830 French controls (mean age 49.1 years; age-range 35.2–61.9 years) were selected in a population-based sample of French subjects that had participated in the Supplementation in Vitamins and Mineral Antioxidants (SU.VI.MAX) study (Hercberg *et al.* 1998).

*Danish replication sample*. The 499 Danish LLIs (male/female ratio 1/3, age range 92-101 years; mean age 96.2 years) were drawn from three population-based nation-wide birth cohort studies conducted at the University of Southern Denmark: The Danish 1905 birth cohort study (Nybo *et al.* 2001), the Danish 1910 birth cohort study (Vestergaard *et al.* 2015, submitted), and the Danish 1915 birth cohort study (Christensen *et al.* 2013). Briefly, the 1905 birth cohort study was initiated in 1998, when participants were 92-93 years of age, and the 1910 and the 1915 Birth Cohort Studies were initiated in 2010, when participants were 100 and 95 years of age, respectively. The 746 younger controls (male/female ratio 2/3; age range 56-71 years; mean age 63.1 years) were drawn from the Study of Middle Aged Danish Twins (MADT) (Skytthe *et al.* 2013). Written informed consent was obtained from all participants and all studies were approved by The Regional Scientific Ethical Committees for Southern Denmark.

**Immunochip genotype calling and quality control**

Initial genotype calling was performed with the GenomeStudio GenTrain 2.0 algorithm (Illumina’s GenomeStudio data analysis software) and the custom generated cluster file of Trynka *et al.* (based on an initial clustering of 2,000 UK samples and subsequent manual readjustment of cluster positions) ([Trynka *et al.* 2011](#_ENREF_13)). SNPs that had >2% missing data, a different missing genotype rates in affected and unaffected individuals (*P_Fisher_*<10^-5^) or deviated from the Hardy-Weinberg equilibrium (exact *P*<10^−4^ in controls) per sample study were excluded using PLINK ([Purcell *et al.* 2007](#_ENREF_11)). Sample quality control measures included sample call rate, overall heterozygosity, relatedness testing and other metrics. Individuals with >2% missing data were removed. Outlier individuals with an average marker heterozygosity of ±5 s.d. away from the sample mean were removed. For robust relatedness testing (IBS/IBD estimation) and population structure analysis, we generated a pruned subset of 20,173 SNPs excluding X- and Y-chromosomes, SNPs in LD (leaving no pairs with r^2^>0.2), and 11 high-LD regions as described by Price et al. ([Price *et al.* 2008](#_ENREF_10)). Pair-wise percentage IBD values were computed using PLINK. By definition, PI_HAT:= P(IBD=2) + 0.5 * P(IBD=1) (proportion IBD). One individual (the one showing greater missingness) from each pair with estimated PI_HAT>0.1875 (which is halfway between expected IBD for third- and second degree relatives) was removed. The remaining 1,458 cases and 6,368 controls were tested for population stratification using the principal components method (PCA) as implemented in SNPRelate ([Zheng *et al.* 2012](#_ENREF_14)). PCA revealed no population outliers (**Supplementary Fig. 3**). 142,136 QCed polymorphic variants and 1,458 German LLI and 6,368 German controls were available for analysis (panel A in **Supplementary Table 1**).

**Statistical analysis in the discovery sample**

Logistic regression was used to perform association analysis. To control for potentially confounding effects due to population stratification, we adjusted for the top ten eigenvectors from PCA in the regression analysis. A quantile-quantile plot of Immunochip association analysis showed a few significant associations in the tail of the distribution (**Supplementary Fig. 4**). Immunochip intensity cluster plots of SNP markers with *P*_Immunochip_<0.01 were manually inspected to ensure that they were well clustered. An associated region was defined by means of PLINK’s clumping procedure using the settings p_1_ ≤ 5×10^-4^, p_2_ ≤ 0.05, r^2^ ≥ 0.5, kb ≤ 250, after cluster plot inspection. Immunochip-wide significance level was defined by Bonferroni correction of the number of LD-independent markers on the Immunochip (0.05/81,248=6.15×10^-7^) using as an estimate for the number of independent markers a pairwise r^2^<0.05 of LD, as was used recently in a previous Immunochip experiment on celiac disease ([Trynka *et al.* 2011](#_ENREF_13)) and in the Cardiochip custom genotyping project ([Musunuru *et al.* 2010](#_ENREF_7); [Lanktree *et al.* 2011](#_ENREF_6)).

For replication, we selected the most strongly associated SNP from each associated region (n=17). Of the 17 SNPs typed in the French and Danish sample two SNPs (rs10500560, rs41280278) failed the genotyping procedure.

**Replication genotyping and statistical analysis**

Follow-up replication genotyping in the French (1,264 LLI and 1,830 younger controls) and Danish study population (499 LLI and 746 younger controls) was carried out using the Sequenom iPlex Mass ARRAY platform (Sequenom, San Diego, CA) (panel B in **Supplementary Table 1**). Quality control was done for each country population separately. Individuals with >5% missing data were removed. SNPs that had >5% missing data or deviated from the Hardy-Weinberg equilibrium (exact *P*<10^−4^ in controls) per sample population were excluded. *P*-values for allele-based tests of phenotypic association for each single replication population were calculated using PLINK. Adjustment for multiple testing was performed by Bonferroni correction for 15 SNPs. PLINK’s fixed-effects meta-analysis function was used to obtain *P*-values for the combined replication data set (*P*_Repl_) (panel B in **Supplementary Table 1**) and the combined discovery-replication data set (*P*_Immunochip+Repl_) (panel A and B in **Supplementary Table 1**).

**Annotation of association boundaries**

LD regions (association boundaries) around lead SNPs were defined by extending in both directions a distance of 0.1 centimorgans (cM). For each locus, candidate genes within regions are listed in columns labeled ‘Key genes’ in **Table 1**.

**Annotation of association with other phenotypes**

Overlaps with other phenotypes were annotated with the National Human Genome Research Institute (NHGRI) GWAS catalog (www.genome.gov/gwastudies, accessed July 15, 2015). All known associations with *P*<5×10^−8^ with any disease or primary phenotype were included. For each longevity locus with association boundaries defined in **Table 1**, we annotated all phenotypes that had at least one associated SNP within the region.

**Supplementary Tables**

**Supplementary Table 1. LLI/control panels used in the analysis.**

**Panel A:** German longevity discovery panel (Immunochip analysis) after quality control. ‘Group’ refers to the scientific group that provided samples. LLI: Long-lived individuals.

| Country | Group | #LLI | #Controls | Mean age in years [SD] | Platform |
| --- | --- | --- | --- | --- | --- |
| Germany | Research Group for Healthy Aging, Institute of Clinical Molecular Biology, Kiel University | 1458 | - | 99.0 [2.6] | Immunochip |
| Germany | Popgen, Kiel University | - | 2481 | 58.3 [15.8] | Immunochip |
| Germany | KORA (Helmholtz Center Munich) | - | 1879 | 56.1 [13.3] | Immunochip |
| Germany | Institute of Medical Informatics, Biometry and Epidemiology, LMU Munich | - | 306 | 47.5 [9.2] | Immunochip |
| Germany | Department of Gastroenterology, Hepatology and Endocrinology, Charité - Universitätsmedizin Berlin | - | 202 | 53.2 [15.7] | Immunochip |
| Germany | Life & Brain Center at the University Clinic in Bonn | - | 1500 | 59.0 [7.7] | Immunochip |
| Total |  | 1458 | 6368 |  |  |

**Panel B:** Longevity replication panel after quality control. LLI: Long-lived individuals.

| Country | Group | #LLI | #Controls | Mean age in years [SD] | Platform |
| --- | --- | --- | --- | --- | --- |
| France | CEPH, Paris, France | 1257 | - | 102.4 [3.0] | Sequenom |
| France | Univ. Sorbonne Paris Cité-UREN, INSERM U557, Bobigny, France | - | 1811 | 49.1 [6.2] | Sequenom |
| Denmark | University of Southern Denmark, Denmark | 493 | - | 96.2 [3.1] | Sequenom |
| Denmark | University of Southern Denmark, Denmark | - | 740 | 63.1 [3.7] | Sequenom |
| Total |  | 1750 | 2551 |  |  |

**Supplementary Table 2. Immunochip association statistics in German panel (panel A, Supplementary Table 1) for 84 SNPs with *P*_Immunochip_<5×10^‑4^.**

| **dbSNP ID** | **A1** | **P** | **OR (95% CI)** |
| --- | --- | --- | --- |
| rs2282701 | A | 0.0002867 | 1.32 (1.13-1.52) |
| rs60905519 | T | 0.0004879 | 1.24 (1.01-1.40) |
| rs12561997 | A | 0.0003523 | 1.25 (1.11-1.41) |
| rs6696981 | T | 0.0003700 | 1.25 (1.11-1.41) |
| rs6684479 | T | 0.0004015 | 1.25 (1.10-1.41) |
| rs6426748 | T | 0.0004196 | 1.24 (1.10-1.41) |
| rs199879172 | C | 0.0004328 | 1.24 (1.10-1.40) |
| rs35553251 | T | 0.0003845 | 1.25 (1.10-1.41) |
| rs6695165 | C | 0.0004485 | 1.24 (1.10-1.41) |
| rs7513562 | C | 0.0004227 | 1.25 (1.10-1.41) |
| rs7550872 | C | 0.0004299 | 1.24 (1.10-1.41) |
| rs10888637 | T | 0.0003558 | 1.38 (1.16-1.65) |
| rs6669448 | A | 0.0003821 | 1.38 (1.15-1.64) |
| rs115926530 | T | 0.0001684 | 0.40 (0.25-0.65) |
| rs1729662 | G | 0.0004831 | 1.16 (1.07-1.26) |
| rs7601200 | T | 0.0001292 | 1.17 (1.08-1.27) |
| rs2242102 | A | 0.0004901 | 1.15 (1.07-1.25) |
| rs62195091 | T | 0.0002637 | 1.22 (1.10-1.36) |
| rs12472244 | G | 0.0003768 | 1.19 (1.08-1.32) |
| rs73075944 | C | 0.0001581 | 1.21 (1.10-1.33) |
| rs4839594 | G | 0.0001973 | 1.22 (1.10-1.36) |
| rs77048564 | T | 0.0001724 | 1.47 (1.20-1.80) |
| rs115706262 | A | 0.0001939 | 1.47 (1.20-1.79) |
| rs1906599 | T | 0.0001286 | 0.82 (0.74-0.91) |
| rs3775662 | T | 0.0002152 | 2.76 (1.61-4.73) |
| rs115522986 | A | 0.0001675 | 1.55 (1.23-1.94) |
| rs6878559 | G | 0.0004166 | 0.86 (0.80-0.94) |
| rs2243677 | C | 0.0003120 | 1.19 (1.08-1.31) |
| rs2706348 | A | 0.0003468 | 1.19 (1.08-1.31) |
| rs2706349 | A | 0.0003342 | 1.19 (1.08-1.31) |
| rs2522414 | C | 0.0004152 | 1.19 (1.08-1.30) |
| rs2706353 | A | 0.0004237 | 1.18 (1.08-1.30) |
| rs2706372 | T | 0.0003113 | 1.19 (1.08-1.31) |
| rs6596086 | C | 0.0004089 | 1.18 (1.08-1.30) |
| rs2106984 | A | 0.0004678 | 1.18 (1.08-1.30) |
| rs9465828 | G | 0.0004872 | 5.01 (2.03-12.39) |
| rs7739438 | A | 0.0002349 | 3.87 (1.88-7.95) |
| rs6900398 | C | 0.0002353 | 3.87 (1.88-7.95) |
| rs11764674 | A | 7.763e-05 | 0.62 (0.49-0.79) |
| rs2720658 | T | 0.0004073 | 1.16 (1.07-1.26) |
| rs34266322 | G | 0.0002952 | 1.32 (1.14-1.53) |
| rs8177060 | A | 0.0002873 | 0.62 (0.48-0.80) |
| rs79545610 | G | 0.0001575 | 1.41 (1.18-1.69) |
| rs12248559 | A | 0.0004248 | 1.29 (1.12-1.48) |
| rs34284450 | T | 0.000462 | 0.68 (0.54-0.84) |
| rs78817795 | T | 5.48e-05 | 1.35 (1.17-1.56) |
| rs7893707 | C | 0.0001017 | 1.61 (1.26-2.04) |
| rs372813 | A | 0.0001883 | 0.83 (0.75-0.91) |
| rs11065987 | G | 0.0004807 | 0.86 (0.79-0.94) |
| rs7982347 | C | 0.0004981 | 1.24 (1.10-1.39) |
| rs912128 | A | 0.000268 | 1.27 (1.12-1.44) |
| rs4788547 | A | 2.382e-05 | 0.83 (0.76-0.90) |
| rs12446005 | A | 0.000283 | 1.18 (1.08-1.29) |
| rs1345868 | A | 0.0003045 | 1.18 (1.08-1.28) |
| rs10500560 | A | 0.0002131 | 1.18 (1.08-1.29) |
| rs4788567 | A | 6.504e-06 | 0.81 (0.74-0.89) |
| rs7350856 | T | 6.755e-06 | 0.81 (0.74-0.89) |
| rs1580602 | A | 0.000413 | 0.86 (0.79-0.94) |
| rs39536 | T | 1.36e-05 | 0.83 (0.76-0.90) |
| rs907875 | C | 1.228e-05 | 0.82 (0.75-0.90) |
| rs11078576 | T | 3.718e-06 | 1.21 (1.12-1.31) |
| rs4790791 | T | 8.434e-05 | 1.18 (1.08-1.27) |
| rs9895574 | G | 9.931e-05 | 1.17 (1.08-1.27) |
| rs7209554 | A | 0.0001285 | 0.84 (0.77-0.92) |
| rs375241 | T | 0.0001851 | 1.19 (1.09-1.30) |
| rs319758 | A | 0.0004218 | 1.18 (1.07-1.29) |
| rs17694365 | A | 0.0002236 | 1.23 (1.10-1.36) |
| rs4804633 | G | 6.214e-05 | 1.19 (1.09-1.30) |
| rs28399637 | A | 0.0002622 | 0.85 (0.77-0.93) |
| rs2075650 | G | 7.01e-09 | 0.69 (0.60-0.78) |
| rs10420331 | A | 0.0003131 | 0.86 (0.79-0.93) |
| rs41280278 | C | 1.655e-05 | 1.50 (1.25-1.80) |
| rs3848722 | G | 0.000296 | 1.17 (1.07-1.27) |
| rs73130504 | T | 0.0001221 | 1.40 (1.18-1.66) |
| rs4810482 | C | 0.0004472 | 1.16 (1.07-1.26) |
| rs45521633 | T | 0.0001397 | 1.41 (1.18-1.68) |
| rs6074009 | T | 0.0001021 | 1.22 (1.10-1.34) |
| rs6065921 | G | 9.497e-05 | 1.22 (1.10-1.34) |
| rs56347917 | G | 0.0003265 | 1.20 (1.09-1.32) |
| rs76926652 | T | 0.0002743 | 1.19 (1.08-1.30) |
| rs76659719 | C | 0.0004303 | 1.20 (1.08-1.32) |
| rs17380117 | G | 0.0004926 | 1.19 (1.08-1.31) |
| rs77048809 | A | 0.0004868 | 1.19 (1.08-1.32) |
| rs8130757 | T | 0.0002543 | 1.74 (1.30-2.34) |
| rs2282701 | A | 0.0002867 | 1.32 (1.13-1.52) |
| rs60905519 | T | 0.0004879 | 1.24 (1.01-1.40) |

**dbSNP id:** rs ID; **A1:** minor allele; ; **P/OR:** Nominal P-value (not corrected for multiple testing) and corresponding allelic odds ratio and 95% confidence interval with respect to A1.

**Supplementary Table 3. Immunochip association statistics in German panel (panel A, Supplementary Table 1) for the 15 SNPs selected for replication.**

| **Chr.** | **Position** | **dbSNP ID** | **A1** | **A2** | **AF cases** | **AF controls** | **P** | **OR (95% CI)** |
| --- | --- | --- | --- | --- | --- | --- | --- | --- |
| 1 | 49426459 | rs10888637 | T | C | 0.05898 | 0.04405 | 0.0003558 | 1.38 (1.16-1.65) |
| 4 | 26055272 | rs77048564 | T | C | 0.04552 | 0.03078 | 0.0001724 | 1.47 (1.20-1.80) |
| 5 | 131935477 | rs2706372 | T | C | 0.2565 | 0.2241 | 0.0003113 | 1.19 (1.08-1.31) |
| 13 | 100074381 | rs912128 | A | G | 0.1173 | 0.09446 | 0.000268 | 1.27 (1.12-1.44) |
| 16 | 71634544 | rs4788547 | A | G | 0.3077 | 0.3479 | 2.382e-05 | 0.83 (0.76-0.90) |
| 16 | 71893498 | rs4788567 | A | G | 0.2716 | 0.3138 | 6.504e-06 | 0.81 (0.74-0.89) |
| 16 | 86532032 | rs39536 | T | C | 0.3244 | 0.3673 | 1.36e-05 | 0.83 (0.76-0.90) |
| 17 | 5502661 | rs907875 | C | T | 0.2938 | 0.3350 | 1.228e-05 | 0.82 (0.75-0.90) |
| 17 | 5505774 | rs11078576 | T | C | 0.4852 | 0.4380 | 3.718e-06 | 1.21 (1.12-1.31) |
| 17 | 31546315 | rs375241 | T | C | 0.2740 | 0.2405 | 0.0001851 | 1.19 (1.09-1.30) |
| 19 | 11772989 | rs4804633 | G | T | 0.3443 | 0.3063 | 6.214e-05 | 1.19 (1.09-1.30) |
| 20 | 44646855 | rs45521633 | T | C | 0.0583 | 0.04185 | 0.0001397 | 1.41 (1.18-1.68) |
| 20 | 44714510 | rs6065921 | G | A | 0.2167 | 0.1857 | 9.497e-05 | 1.22 (1.10-1.34) |
| 20 | 44730817 | rs76926652 | T | C | 0.2555 | 0.2243 | 0.0002743 | 1.19 (1.08-1.30) |
| 21 | 16741959 | rs8130757 | T | C | 0.0216 | 0.01264 | 0.0002543 | 1.74 (1.29-2.34) |

**Chr**: chromosome of marker; **Position**: genomic positions were retrieved from NCBI’s dbSNP build v141 (genome build hg19); **dbSNP id:** rs ID; **A1:** minor allele; **A2:** major allele; **AF:** allele frequency of A1; **P/OR:** Nominal P-value (not corrected for multiple testing) and corresponding allelic odds ratio and 95% confidence interval with respect to A1.

**Supplementary Table 4. Association statistics in French and Danish samples (panel B, Supplementary Table 1) for the 15 SNPs selected for replication.**

|  |  |  |  |  | **Replication Denmark (493/740)** | |  |  | **Replication France**  **(1,257/1,811)** | |  |  | **Replication combined**  **(1,750/2,551)** | |
| --- | --- | --- | --- | --- | --- | --- | --- | --- | --- | --- | --- | --- | --- | --- |
| **Chr.** | **Position** | **dbSNP ID** | **A1** | **A2** | **P_Denmark_** | **OR (95% CI)** | **AF cases** | **AF controls** | **P_France_** | **OR (95% CI)** | **AF cases** | **AF controls** | **P_Repl_** | **OR (95% CI)** |
| 1 | 49426459 | rs10888637 | T | C | 0.6813 | 1.09 (0.73-1.62) | 0.04462 | 0.04122 | 0.8855 | 0.98 (0.76-1.27) | 0.04177 | 0.04252 | 0.9199 | 1.01 (0.82-1.25) |
| 4 | 26055272 | rs77048564 | T | C | 0.9636 | 0.99 (0.61-1.59) | 0.02941 | 0.02973 | 0.05929 | 0.71 (0.50-1.02) | 0.01909 | 0.0265 | 0.1237 | 0.80 (0.60-1.06) |
| 5 | 131935477 | rs2706372 | T | C | 0.07594 | 1.19 (0.98-1.45) | 0.2312 | 0.2014 | 2.684×10^-3^ | 1.21 (1.07-1.38) | 0.2189 | 0.1877 | 4.951×10^-4^ | 1.21 (1.09-1.34) |
| 13 | 100074381 | rs912128 | T | C | 0.3915 | 0.89 (0.68-1.16) | 0.09736 | 0.1081 | 0.4733 | 0.94 (0.78-1.12) | 0.08559 | 0.09088 | 0.2832 | 0.92 (0.79-1.07) |
| 16 | 71681153 | rs39536 | A | G | 0.6947 | 1.03 (0.87-1.22) | 0.3618 | 0.3541 | 0.369 | 1.05 (0.94-1.17) | 0.3429 | 0.3319 | 0.3302 | 1.05 (0.96-1.15) |
| 16 | 71893498 | rs4788547 | A | G | 0.05649 | 1.18 (1.00-1.39) | 0.3884 | 0.3507 | 0.643 | 1.03 (0.92-1.15) | 0.3018 | 0.2962 | 0.1477 | 1.07 (0.98-1.17) |
| 16 | 86532032 | rs4788567 | A | G | 0.1114 | 1.15 (0.97-1.36) | 0.3428 | 0.3122 | 0.6755 | 1.03 (0.91-1.15) | 0.2800 | 0.2752 | 0.2178 | 1.06 (0.97-1.17) |
| 17 | 5502661 | rs11078576 | T | C | 0.9587 | 1.00 (0.85-1.18) | 0.4381 | 0.4371 | 0.06668 | 1.10 (0.99-1.22) | 0.4379 | 0.4144 | 0.1154 | 1.07 (0.98-1.17) |
| 17 | 5505774 | rs375241 | T | C | 0.2718 | 0.90 (0.75-1.08) | 0.2596 | 0.2797 | 0.353 | 0.94 (0.83-1.07) | 0.2172 | 0.2272 | 0.1666 | 0.93 (0.84-1.03) |
| 17 | 31546315 | rs907875 | G | A | 0.9598 | 1.00 (0.85-1.19) | 0.3523 | 0.3514 | 0.03388 | 0.89 (0.80-0.99) | 0.3266 | 0.3527 | 0.07799 | 0.92 (0.84-1.01) |
| 19 | 11772989 | rs4804633 | G | T | 0.4775 | 0.94 (0.79-1.12) | 0.2927 | 0.3061 | 0.8042 | 1.01 (0.91-1.13) | 0.2971 | 0.2942 | 0.8626 | 0.99 (0.90-1.09) |
| 20 | 44646855 | rs45521633 | T | C | 0.6367 | 0.91 (0.61-1.35) | 0.04260 | 0.04662 | 0.7602 | 1.04 (0.82-1.31) | 0.05171 | 0.04997 | 0.9797 | 1.00 (0.82-1.22) |
| 20 | 44714510 | rs6065921 | G | A | 0.7888 | 1.03 (0.85-1.25) | 0.2201 | 0.2155 | 0.6959 | 0.97 (0.85-1.11) | 0.1842 | 0.1881 | 0.8602 | 0.99 (0.89-1.10) |
| 20 | 44730817 | rs76926652 | T | C | 0.8138 | 0.98 (0.81-1.18) | 0.2525 | 0.2568 | 0.7655 | 0.98 (0.87-1.11) | 0.2281 | 0.2314 | 0.7055 | 0.98 (0.89-1.09) |
| 21 | 16741959 | rs8130757 | T | C | 0.2783 | 1.43 (0.75-2.74) | 0.01826 | 0.01284 | 0.2321 | 0.77(0.51-1.18) | 0.01352 | 0.01739 | 0.6783 | 0.93 (0.65-1.32) |

**Chr**: chromosome of marker; **Position**: genomic positions were retrieved from NCBI’s dbSNP build v141 (genome build hg19); **dbSNP id:** rs ID; **A1:** minor allele; **A2:** major allele; **AF:** allele frequency of A1; **P/OR:** Nominal P-value (not corrected for multiple testing) and corresponding allelic odds ratio and 95% confidence interval with respect to minor allele. For each panel, numbers of longevity cases/controls are displayed in parentheses.

# Supplementary Figures

**Supplementary Figure 1. Manhattan plot of Immunochip association statistics of 142,136 SNPs.** Red horizontal line indicates an Immunochip-wide significance level of 6.15×10^-7^. Black horizontal line indicates our selected *P*-value threshold for replication (5×10^-4^).


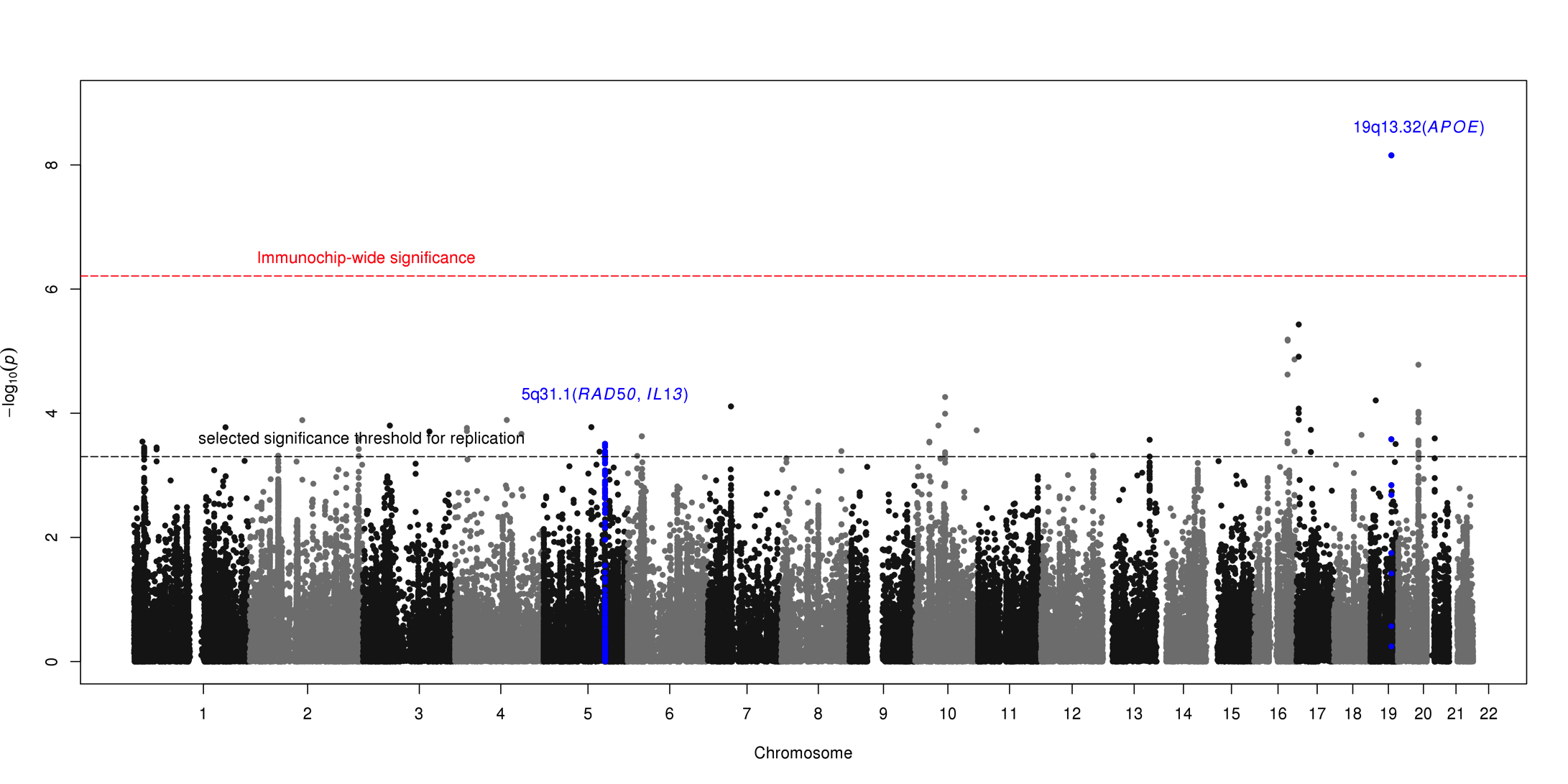


**Supplementary Figure 2.**

**(a)**

**(b)**

**(c)**

Regional association plots (from Immunochip analysis; panel A in **Supplementary Table 1)** of established longevity susceptibility loci. (**a**) 19q13 at *APOE* (**b**) 5q33.3 at *EBF1* and (**c**) 6q21 at *FOXO3*. All loci are sparsely covered on the Immunochip.

**Supplementary Figure 3. Principal component analysis of QCed Immunochip data.**

**(a)** The first two principal components of all Immunochip samples (panel A in **Supplementary Table 1**) are shown. Samples are colored by genotyping batch code. **(b)** Even distribution of cases and controls along the first six principal components stratified by case/control. AGI1: German LLI samples; POPCOF1/GCON1/BSPSCP1: German control samples from the popgen biorepository; HNR1: German control samples from the Heinz Nixdorf Recall study; KORA1/KORA2: German control samples from the KORA study; Pittsburgh1: German control samples recruited by the Bavarian Red Cross and the Charité-Universitätsmedizin Berlin genotyped in Pittsburgh.

**(a)**


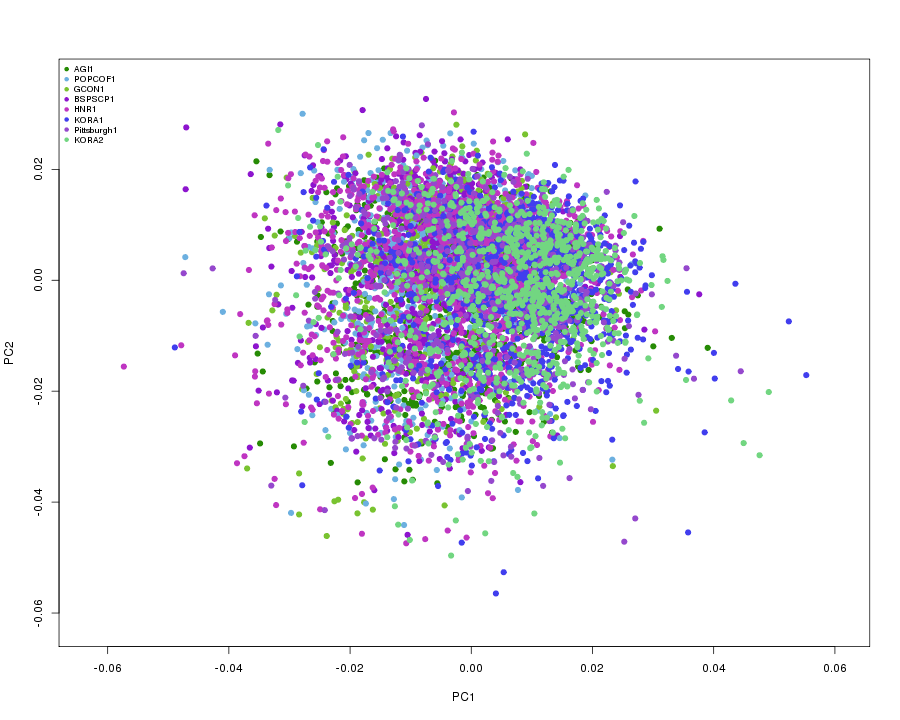


**(b)**


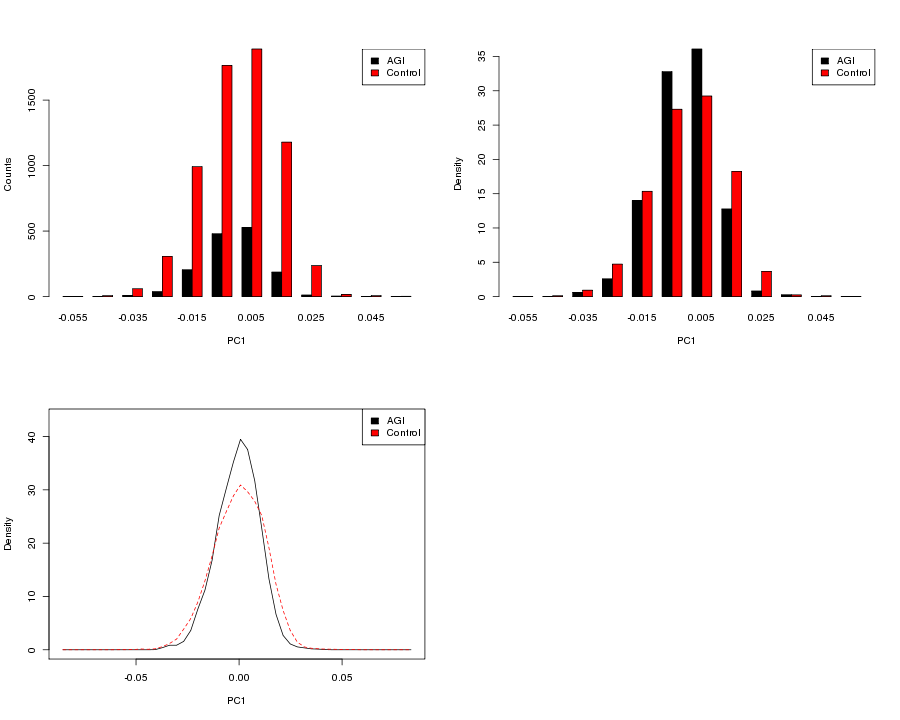


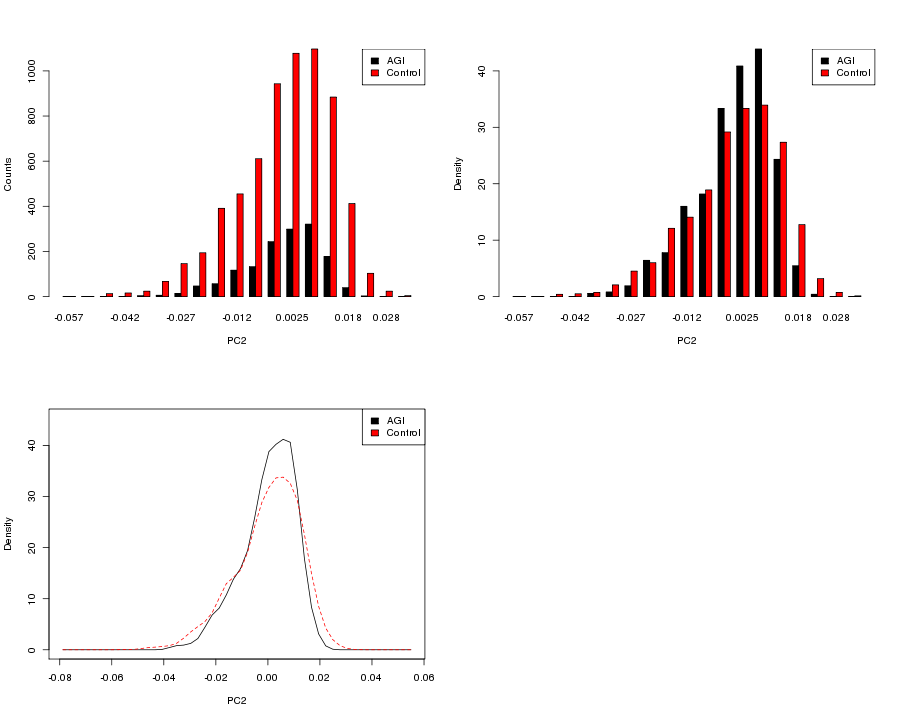


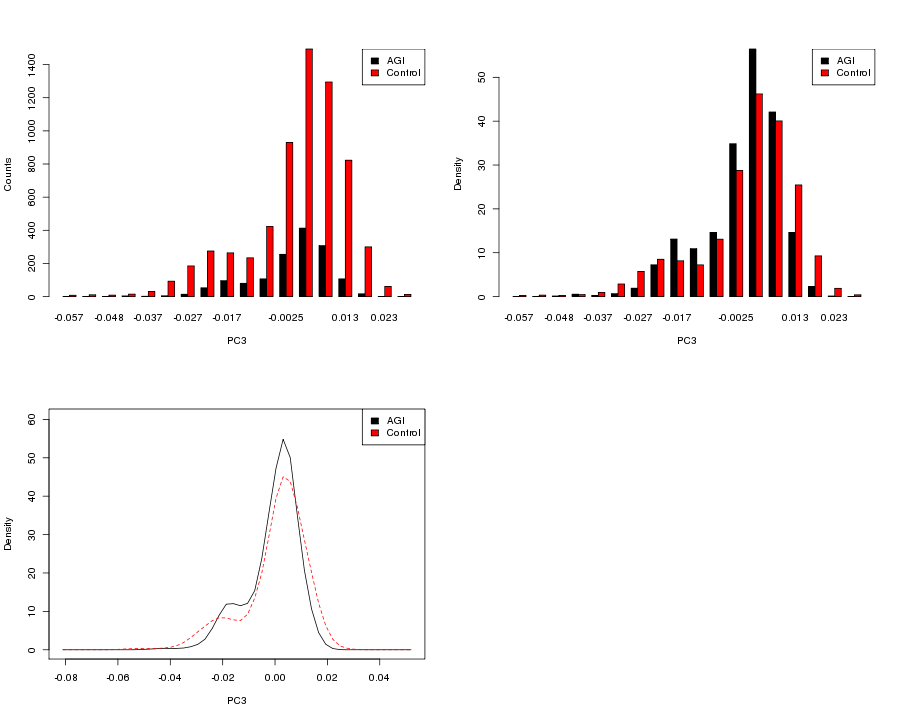


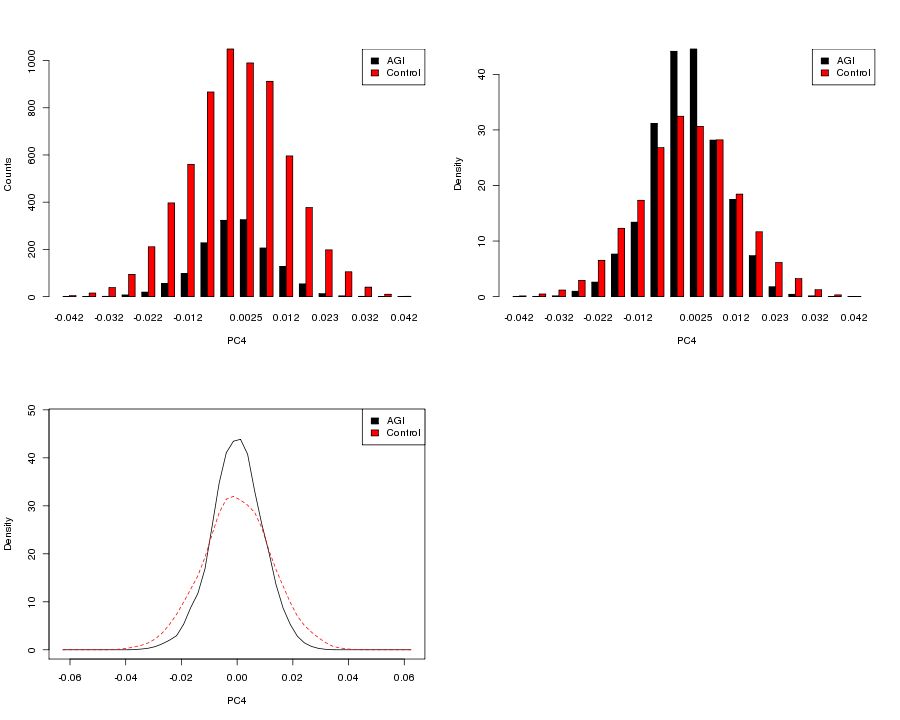


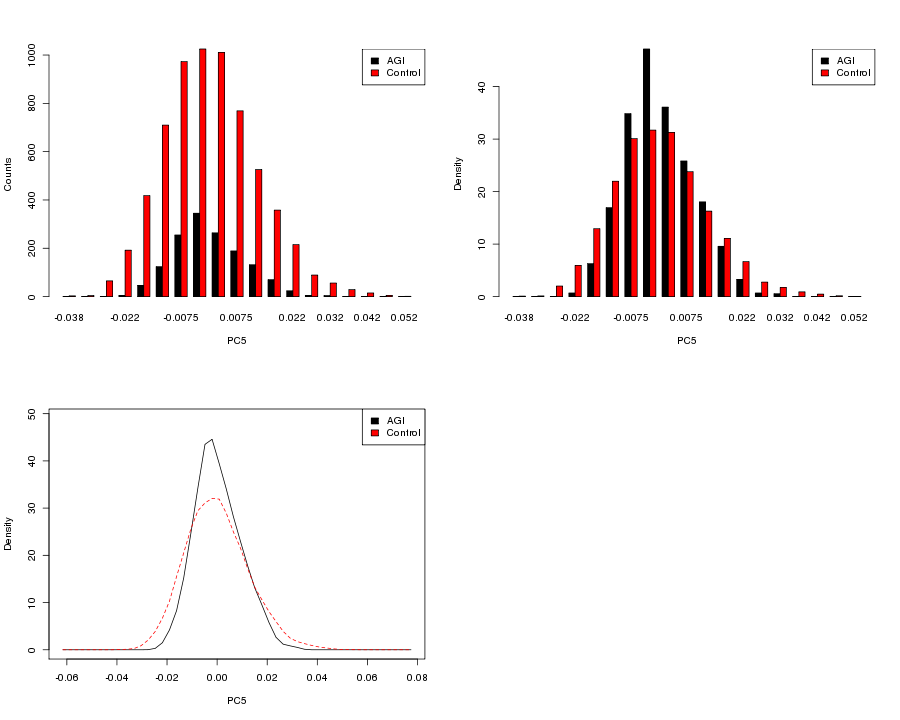


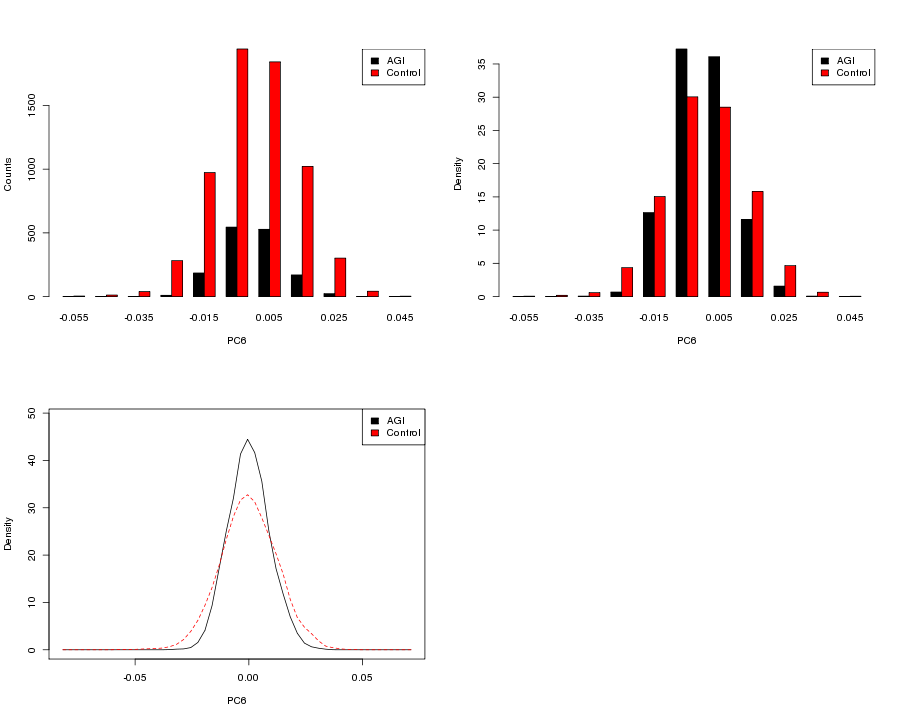


**Supplementary Figure 4**. Quantile-quantile (Q-Q) plot for the discovery panel (panel A in **Supplementary Table 1**). Only markers that passed the quality criteria were used for plotting. The 2.5th and 97.5th percentiles of the distribution under random sampling and the null hypothesis of no association form the 95% concentration band. The genomic inflation factor λ is defined as the ratio of the medians of the sample χ2 test statistics and the 1-d.f. χ2 distribution (0.455) ([Devlin & Roeder 1999](#_ENREF_2)). Since the estimated genomic inflation factor λ scales with sample size, it is informative to report the inflation factor for an equivalent study of 1000 cases and 1000 controls (λ_1000_) by rescaling λ ([de Bakker *et al.* 2008](#_ENREF_1)). Estimated genomic inflation factor is λ=0.983 (λ_1000_=0.993) from Immunochip association analysis.


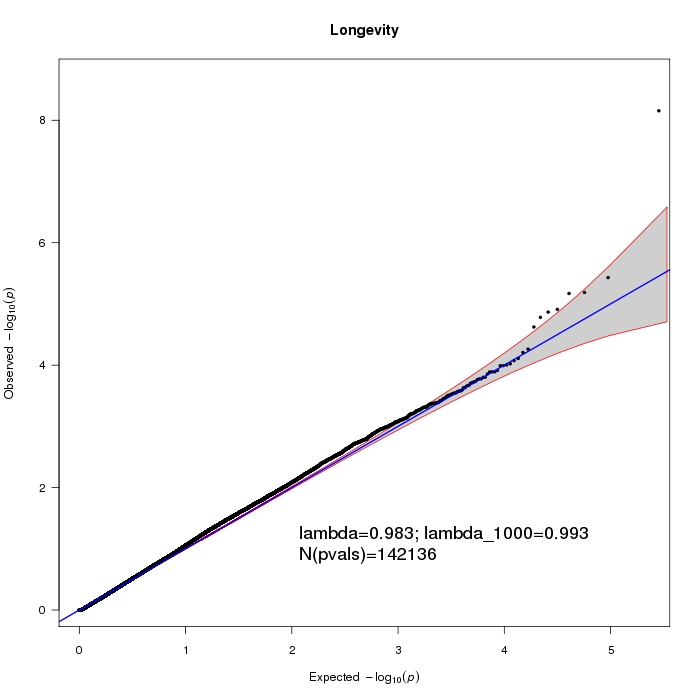


**Supplementary References**

Blanché H, Cabanne L, Sahbatou M, Thomas G (2001). A study of French centenarians: are ACE and APOE associated with longevity? *CR Acad Sci III.* **324**, 129-135.

Christensen K, Thinggaard M, Oksuzyan A, Steenstrup T, Andersen-Ranberg K, Jeune B, McGue M , Vaupel JW (2013). Physical and cognitive functioning of people older than 90 years: a comparison of two Danish cohorts born 10 years apart. *Lancet*. **382**, 1507-1513.

de Bakker PI, Ferreira MA, Jia X, Neale BM, Raychaudhuri S, Voight BF (2008). Practical aspects of imputation-driven meta-analysis of genome-wide association studies. *Hum Mol Genet*. **17**, R122-128.

Devlin B, Roeder K (1999). Genomic control for association studies. *Biometrics*. **55**, 997-1004.

Flachsbart F, Caliebe A, Kleindorp R, Blanche H, von Eller-Eberstein H, Nikolaus S, Schreiber S, Nebel A (2009). Association of FOXO3A variation with human longevity confirmed in German centenarians. *Proc Natl Acad Sci U S A*. **106**, 2700-2705.

Holle R, Happich M, Lowel H, Wichmann HE (2005). KORA--a research platform for population based health research. *Gesundheitswesen*. **67 Suppl 1**, S19-25.

Krawczak M, Nikolaus S, von Eberstein H, Croucher PJ, El Mokhtari NE, Schreiber S (2006). PopGen: population-based recruitment of patients and controls for the analysis of complex genotype-phenotype relationships. *Community Genet*. **9**, 55-61.

Lanktree MB, Guo Y, Murtaza M, Glessner JT, Bailey SD, Onland-Moret NC, Lettre G, Ongen H, Rajagopalan R, Johnson T, Shen H, Nelson CP, Klopp N, Baumert J, Padmanabhan S, Pankratz N, Pankow JS, Shah S, Taylor K, Barnard J, Peters BJ, Maloney CM, Lobmeyer MT, Stanton A, Zafarmand MH, Romaine SP, Mehta A, van Iperen EP, Gong Y, Price TS, Smith EN, Kim CE, Li YR, Asselbergs FW, Atwood LD, Bailey KM, Bhatt D, Bauer F, Behr ER, Bhangale T, Boer JM, Boehm BO, Bradfield JP, Brown M, Braund PS, Burton PR, Carty C, Chandrupatla HR, Chen W, Connell J, Dalgeorgou C, Boer A, Drenos F, Elbers CC, Fang JC, Fox CS, Frackelton EC, Fuchs B, Furlong CE, Gibson Q, Gieger C, Goel A, Grobbee DE, Hastie C, Howard PJ, Huang GH, Johnson WC, Li Q, Kleber ME, Klein BE, Klein R, Kooperberg C, Ky B, Lacroix A, Lanken P, Lathrop M, Li M, Marshall V, Melander O, Mentch FD, Meyer NJ, Monda KL, Montpetit A, Murugesan G, Nakayama K, Nondahl D, Onipinla A, Rafelt S, Newhouse SJ, Otieno FG, Patel SR, Putt ME, Rodriguez S, Safa RN, Sawyer DB, Schreiner PJ, Simpson C, Sivapalaratnam S, Srinivasan SR, Suver C, Swergold G, Sweitzer NK, Thomas KA, Thorand B, Timpson NJ, Tischfield S, Tobin M, Tomaszewski M, Verschuren WM, Wallace C, Winkelmann B, Zhang H, Zheng D, Zhang L, Zmuda JM, Clarke R, Balmforth AJ, Danesh J, Day IN, Schork NJ, de Bakker PI, Delles C, Duggan D, Hingorani AD, Hirschhorn JN, Hofker MH, Humphries SE, Kivimaki M, Lawlor DA, Kottke-Marchant K, Mega JL, Mitchell BD, Morrow DA, Palmen J, Redline S, Shields DC, Shuldiner AR, Sleiman PM, Smith GD, Farrall M, Jamshidi Y, Christiani DC, Casas JP, Hall AS, Doevendans PA, Christie JD, Berenson GS, Murray SS, Illig T, Dorn GW, 2nd, Cappola TP, Boerwinkle E, Sever P, Rader DJ, Reilly MP, Caulfield M, Talmud PJ, Topol E, Engert JC, Wang K, Dominiczak A, Hamsten A, Curtis SP, Silverstein RL, Lange LA, Sabatine MS, Trip M, Saleheen D, Peden JF, Cruickshanks KJ, Marz W, O'Connell JR, Klungel OH, Wijmenga C, Maitland-van der Zee AH, Schadt EE, Johnson JA, Jarvik GP, Papanicolaou GJ, Hugh Watkins on behalf of P, Grant SF, Munroe PB, North KE, Samani NJ, Koenig W, Gaunt TR, Anand SS, van der Schouw YT, Meena Kumari on behalf of the Whitehall IIS, the WKG, Soranzo N, Fitzgerald GA, Reiner A, Hegele RA, Hakonarson H, Keating BJ (2011). Meta-analysis of Dense Genecentric Association Studies Reveals Common and Uncommon Variants Associated with Height. *Am J Hum Genet*. **88**, 6-18.

Musunuru K, Lettre G, Young T, Farlow DN, Pirruccello JP, Ejebe KG, Keating BJ, Yang Q, Chen MH, Lapchyk N, Crenshaw A, Ziaugra L, Rachupka A, Benjamin EJ, Cupples LA, Fornage M, Fox ER, Heckbert SR, Hirschhorn JN, Newton-Cheh C, Nizzari MM, Paltoo DN, Papanicolaou GJ, Patel SR, Psaty BM, Rader DJ, Redline S, Rich SS, Rotter JI, Taylor HA, Jr., Tracy RP, Vasan RS, Wilson JG, Kathiresan S, Fabsitz RR, Boerwinkle E, Gabriel SB, Resource NCGA (2010). Candidate gene association resource (CARe): design, methods, and proof of concept. *Circulation. Cardiovascular genetics*. **3**, 267-275.

Nebel A, Croucher PJ, Stiegeler R, Nikolaus S, Krawczak M, Schreiber S (2005). No association between microsomal triglyceride transfer protein (MTP) haplotype and longevity in humans. *Proceedings of the National Academy of Sciences of the United States of America*. **102**, 7906-7909.

Nebel A, Kleindorp R, Caliebe A, Nothnagel M, Blanche H, Junge O, Wittig M, Ellinghaus D, Flachsbart F, Wichmann HE, Meitinger T, Nikolaus S, Franke A, Krawczak M, Lathrop M, Schreiber S (2011). A genome-wide association study confirms APOE as the major gene influencing survival in long-lived individuals. *Mech Ageing Dev*. **132**, 324-330.

Price AL, Weale ME, Patterson N, Myers SR, Need AC, Shianna KV, Ge D, Rotter JI, Torres E, Taylor KD, Goldstein DB, Reich D (2008). Long-range LD can confound genome scans in admixed populations. *Am J Hum Genet*. **83**, 132-135; author reply 135-139.

Purcell S, Neale B, Todd-Brown K, Thomas L, Ferreira MA, Bender D, Maller J, Sklar P, de Bakker PI, Daly MJ, Sham PC (2007). PLINK: a tool set for whole-genome association and population-based linkage analyses. *Am J Hum Genet*. **81**, 559-575.

Schmermund A, Mohlenkamp S, Stang A, Gronemeyer D, Seibel R, Hirche H, Mann K, Siffert W, Lauterbach K, Siegrist J, Jockel KH, Erbel R (2002). Assessment of clinically silent atherosclerotic disease and established and novel risk factors for predicting myocardial infarction and cardiac death in healthy middle-aged subjects: rationale and design of the Heinz Nixdorf RECALL Study. Risk Factors, Evaluation of Coronary Calcium and Lifestyle. *Am Heart J*. **144**, 212-218.

Trynka G, Hunt KA, Bockett NA, Romanos J, Mistry V, Szperl A, Bakker SF, Bardella MT, Bhaw-Rosun L, Castillejo G, de la Concha EG, de Almeida RC, Dias KR, van Diemen CC, Dubois PC, Duerr RH, Edkins S, Franke L, Fransen K, Gutierrez J, Heap GA, Hrdlickova B, Hunt S, Izurieta LP, Izzo V, Joosten LA, Langford C, Mazzilli MC, Mein CA, Midah V, Mitrovic M, Mora B, Morelli M, Nutland S, Nunez C, Onengut-Gumuscu S, Pearce K, Platteel M, Polanco I, Potter S, Ribes-Koninckx C, Ricano-Ponce I, Rich SS, Rybak A, Santiago JL, Senapati S, Sood A, Szajewska H, Troncone R, Varade J, Wallace C, Wolters VM, Zhernakova A, Thelma BK, Cukrowska B, Urcelay E, Bilbao JR, Mearin ML, Barisani D, Barrett JC, Plagnol V, Deloukas P, Wijmenga C, van Heel DA (2011). Dense genotyping identifies and localizes multiple common and rare variant association signals in celiac disease. *Nat Genet*. **43**, 1193-1201.

Zheng X, Levine D, Shen J, Gogarten SM, Laurie C, Weir BS (2012). A high-performance computing toolset for relatedness and principal component analysis of SNP data. *Bioinformatics*. **28**, 3326-3328.
